# Lung tumour cell lines synthesizing peptide hormones established from tumours of four histological types: characterization of the cell lines and analysis of their peptide hormone production.

**DOI:** 10.1038/bjc.1985.132

**Published:** 1985-06

**Authors:** W. Luster, C. Gropp, H. F. Kern, K. Havemann

## Abstract

**Images:**


*Br. J. Cancer* (1985), **51**, 865–875

### Lung tumour cell lines synthesizing peptide hormones established from tumours of four histological types: Characterization of the cell lines and analysis of their peptide hormone production

W. Luster, C. Gropp, H.F. Kern[1] & K. Havemann

*Zentrum für Innere Medizin, Abt. Hämatologie, Marburg; [1]Institut für Zytobiologie, Marburg, FRG.*

**Summary** Thirty permanent and more than 60 primary tumour cell lines were established from pleural and pericardial exudates or wedge biopsies from human bronchial carcinoma. The permanent cell lines have their origin in 6 small cell, 5 large cell, 9 squamous and 5 adeno carcinomas of the lung. Tumour cells were purified from non tumour cells by direct cloning in fluid cultures or by soft agar cloning. *In vitro* secretion of ACTH, bombesin, calcitonin, and neurotensin was demonstrated for lung tumour cells belonging to the four major histological types. Cell suspensions of peptide hormone secreting permanent cell cultures were grown to solid tumours after xenotransplantation into nude mice. Comparative ultrastructural examination of the primary tumour and of cells grown in tissue culture and in xenografts demonstrated the preservation of most tumour type specific structural criteria in the *ex vivo/in vitro* systems. The precent data show that not only tumour cells from small cell carcinoma but also from other histological types are capable of synthesizing a broad spectrum of immunoreactive peptide hormones. This result might be interpreted as indicating a common expression of hormone biosynthesis and secretion by all lung tumours.

The biosynthesis and secretion of peptide hormones by tumour cells from small cell carcinoma of the lung *in vivo* and *in vitro* has been reported by several authors (Berson & Yallow, 1966; Horai *et al.*, 1973; Silva *et al.*, 1974). In some cases lung tumours of this histological type are associated with paraneoplastic syndromes caused by the peptide hormone production of the tumour. In this connection the value of serum ACTH and calcitonin as a tumour marker has been examined during therapy (Roos *et al.*, 1980; Silva *et al.*, 1979) and at diagnosis (Wolfsen & Odell, 1979) of small cell lung cancer. Biochemical examinations of patient sera and fresh tumour tissue resulted in the detection of high molecular weight peptide hormone immunoreactivities probably prohormones (Luster *et al.*, 1982). These early findings led to the assumption that the *in vivo* secretion of calcitonin or even peptide hormone biosynthesis is a specific function of small cell lung tumours (Silva *et al.*, 1979; Moody *et al.*, 1981). Roos *et al.* (1980) supposed the slightly elevated serum calcitonin found in patients with large cell or squamous lung carcinoma to be secondary. Moody *et al.* (1981) demonstrated the *in vitro* biosynthesis of peptide hormones especially bombesin by cell lines derived from small cell lung tumours in contrast to non small cell lung tumours which did not produce comparable amounts of bombesin. The authors concluded that the biosynthesis of bombesin is specific for small cell lung carcinomas.

The present results give evidence for the biosynthesis of immunoreactive ACTH, bombesin, calcitonin, and neurotensin, by lung tumours of different histological types.

### Materials and methods

### *Cell culture methods*

Permanent cell cultures were established from small cell, large cell, squamous and adeno carcinoma of the lung (Kreyberg *et al.*, 1967). All purification steps leading to a permanent cell line were controlled by cytological analysis (Takahasi, 1981). Cell lines of small cell carcioma were derived from pleural or pericardial exudates. Specimens were collected with an anticoagulant, centrifuged and then separated from erythrocytes and cell debris by ficoll gradient (Pharmacia Uppsala, Sweden) centrifugation for 30 min at 800 *g*. The fraction containing the tumour cells (Takahasi, 1981) was carefully collected and washed in a 10-fold volume of MEM Dulbecco's or RPMI-1640 cell culture medium (Boehringer Mannheim, FRG). The cells

Correspondence: W. Luster.
Received 21 January 1985; and in revised form 1 March 1985.

© The Macmillan Press Ltd., 1985

were resuspended in the same medium containing 16.6% foetal calf serum (Paesel Frankfurt, FRG), 0.05 g ml$^{-1}$ streptomycin, 50,000 iu penicillin and 250 $\mu$g ml$^{-1}$ amphotericin (Boehringer Mannheim, FRG) diluted to $10^5$ cells ml$^{-1}$ and plated into cell culture flasks (Nunc Roskilde, Denmark). Non small cell lung tumour cell lines were established from surgically obtained fresh tumour tissue. Solid tissue specimens were washed with antibiotics (0.5 g streptomycin, 50,000 iu penicillin and 250 $\mu$g amphotericin B ml$^{-1}$ PBS) and minced into 1 – 3 mm$^3$ pieces. To obtain a cell suspension the material was disintegrated by collagenase (0.5 mg ml$^{-1}$ PBS, Boehringer Mannheim, FRG) 3 times for 15 min at 20°C. In the case of incomplete disintegration after this procedure an incubation in the presence of 0.5 mg ml$^{-1}$ collagenase and 0.5% trypsin (Boehringer Mannheim, FRG) was followed. Washing and culturing of the tumour cells was performed as described above for the small cell tissue cultures.

After growth of the cells to confluency or to 1 to $5 \times 10^6$ cells per culture flask the medium was analyzed for peptide hormone content. The positive cell cultures were cloned in microplates, analysed for peptide hormones and recloned. If necessary such clones were submitted to an additional purification by soft agar cloning.

### *Soft agar cloning*

Agar (Bacto agar, Difco Detroit, USA) 0.5% in cell culture medium was poured into 35 mm dishes with gas-permeable support (Petriperm, Heraeus, Hanau, FRG) and allowed to harden. Cells $10^4$ –$10^5$ were suspended in 0.3% agar solution and added onto the base layer. Isolated colonies were harvested 3 to 6 weeks later and grown in microplates.

### *Xenotransplantation in athymic nude mice*

Malignant exudates or fresh tumour tissue were treated as described above and instead of seeding into culture flasks cell suspensions were injected s.c. into athymic nude mice (NMRI). Cell suspensions from permanent cell lines were submitted to the same procedure. All handling of mice took place in a laminar flow hood (BH-26 TG, Flow GmbH, Meckenheim, FRG). Mice were maintained in sterile plastic cases in a standard animal room (37°C; 70% humidity of the atmosphere; "Luftstromisolator 3/30", Altromin Lage, FRG). Once tumours reached a volume >4 cm$^3$, the tumours were transplanted into new mice or prepared for cell culture and histological examination.

### *Cytological methods*

*Cytodiagnostics* Cell suspensions were centrifuged directly on a microscopical slide (cytocentrifuge: Heraeus Hanau, FRG). Cells were stained according to Pappenheim (Henning, 1966) and analyzed for tumour cells with a light microscope (Takahasi, 1981) Carbohydrates were demonstrated by periodic acid Schiff's reaction (Barck, 1982) not only in single cells but also in paraffin or bouin-fixed tumour sections.

*Electron microscopy* Specimens of the original tumour biopsies, from xenografts and from cell lines were fixed by immersion in 2.5% glutaraldehyde and 2% freshly prepared formaldehyde buffered in 0.1 M cacodylate at pH 7.4. After postfixation in 1% osmium tetroxide and dehydration in a graded series of alcohol, tissue, samples were embedded in Epon (Serva, Heidelberg). From each tumour 6 –8 blocks were analyzed first by light microscopy using semithin sections (0.5 $\mu$m) stained with 1% Azur II and suitable areas were selected for thin sections which were stained with 1% uranylacetate and lead citrate (Reynolds, 1963). They were examined in a ZEISS EM 9S electron microscope.

### *Measurement of peptide hormones*

ACTH, CA 19-9, TPA (IDW Dreieich, FRG), calcitonin, bombesin, and neurotensin (Immunonuclear Minnesota, USA), $\beta$-lipotropin (NEN Chemicals New brunswich, USA), ferritin (Behringwerke AG Marburg, FRG), $\beta$-HCG (IRE, Brussels Belgium), CEA (Abbot Dreieich, FRG), oestriol, cortisol, progesterone, testosterone (Biermann GmbH Bad Nauheim, FRG), were determined by commercially available radioimmunoassays. The assay of substance P was accomplished according to McIntosh *et al.* (1978). The specificity of the hormone determinations was controlled by displacement of the radioactive labelled hormone by tumour cell culture medium and synthetic hormone or hormone preparations. For evaluation of the reliability of the commercially available assays the profiles of tumour cell culture medium dilution curves in fresh cell culture medium and dilution of synthetic hormone or hormone preparations were compared.

### Results

### *Growth of lung tumour cells* in vitro

Thirty permanent tumour cell lines were established from tumour tissue or exudates from more than

100 patients with small cell, large cell, squamous and adeno carcinoma of the lung (MR-1-MR-109). The cell lines have been stable for 12 -24 months.

Best results in primary culture of the tumour cells were accomplished after mincing the tissue into small pieces followed by enzyme disintegration. Mechanical or enzyme disintegration alone led to a distinctly lower yield of vital tumour cells. More than 90% of the fresh tumour material was grown in primary suspension cultures. Half of these cell cultures stopped growing within a maximum period of 3 months and finally died. Some tumour cells were observed which were obviously dependent on a co-culture with fibroblasts. In the presence of human or mouse fibroblasts such cells exhibited growth rates similar to those of optimally growing permanent cell lines of the same histology. After removal of the fibroblasts the cell cultures died within a maximum of 3 passages. In addition to the fibroblasts tumour cell cultures were contaminated by pleural epithelial cells, macrophages, lymphocytes, and erythrocytes. These cells, however, disappeared within 3 to 4 culture passages.

Tumour cell cultures were purified by soft agar cloning, if a significant decrease in contamination was not observed after 3 passages.

The successful establishment of permanent tumour cell lines was shown to be dependent on the number of cells and the condition of the original tumour tissue. At least $2 \times 10^9$ vital tumour cells free from a large number of dead cells were necessary for easy establishment of a permanent cell line. In some cases of small cell lung carcinoma xeno-transplantation of the fresh tumour cells improved the rate of tumours growing in permanent culture. Cell lines from small cell lung tumours grew in suspension cultures, as floating cell aggregates as well as adherent to plastic. In some cases adherent growth began but not before the fifth passage of the cells: Floating cell aggregates attached to the plastic surface and the cells switched to adherent growth. Tumour cell lines derived from large cell, squamous or adeno carcinoma of the lung were grown in adherent culture with only one exception (MR-13). The growth rate of those cell lines was distinctly higher than the growth rate of the small cell suspension cultures. It increased significantly within the first 5 culture passages. During primary culture cells grew to confluency within 1 to 4 weeks, the 5th passage took only 3 to 10 days.

All permanent lung tumour cell lines exhibited morphological stability for more than 15 passages. Vital cells were examined for morphological alterations by phase contrast microscopy. During the first passages adherent growing cell lines developed a spindle form of the single cell. Cytological alterations, however, were not observed. Cloning efficiency in 0.3% soft agar was examined in parallel with each passage in some examples. No significant change was observed during the first 15 passages. Cloning efficiency ranged from 0.003 -0.2%. The soft agar growth rate of small cell lung tumour cells was $\sim 10$ fold higher than the growth of non small cell lung tumour cells.

Tumour cell suspensions which had been frozen at $-196°C$ and stored at $-130°C$ were recultured with a viability of $>90\%$ of the frozen cells. Morphological differences between cells from a continuously passaged permanent cell line and the recultured frozen cells were not observed.

*Xenotransplantation of permanent lung tumour cell culture*

Tumour cells from permanent lung tumour cell lines were injected s.c. into NMRI nu/nu mice. The development of a tumour in the mouse dependent on the number of tumour cells injected and on the age of the animal. Best results were obtained with the injection of $1\text{–}2 \times 10^7$ tumour cells into mice which were almost 4 weeks old. As a rule the transplanted tumour cells developed a solid tumour with the histological characteristics of the original human carcinoma within 4 -14 weeks after injection into the nude mouse. The histological diagnosis was performed routinely by common pathological methods according to the WHO classification. Small cell lung tumour cells developed a solid tumour in the nude mouse distinctly earlier (min. 4 weeks) than non small cell lung tumour cells (max. 14 weeks). Moreover, xenotransplants from small cell lung tumours exhibited a shorter doubling time of growth than non small cell tumours (Table I).

Adherent growing tumour cells which had changed their morphology to a spindle form during passage also developed a mouse tumour with the histology of the original human tumour. After disintegration of the mouse tumour and re-establishment of suspension cultures these cells exhibited the same morphological and cytological features as the primary cultured cells. After several passages in suspension culture the tumour cells redeveloped their spindle configuration while the cytological characteristics were stable.

All xenotransplanted cells from permanent lung tumour cell lines showed, after formation of a solid tumour and suspension reculture, identical morphology, cytology and growth behaviour to that before transplantation. For the non small cell lung tumour cell line MR-13 which, exceptionally, was growing in floating cell aggregates the same behaviour was observed after xenotransplantation.

**Table I** Characteristics of permanent lung tumour cell lines[a]

| *Lung cancer cell line* | | *Origin* | *Doubling time (days) culture* | *Doubling time (days) mouse* | *Tumour growth, mouse $10^7$ cells s.c.* | *Hormones produced* |
|---|---|---|---|---|---|---|
| Small cell carcinoma | MR-22 | Pleural fluid | 4 | 2–7 | 14 | bombesin, calcitonin, neurotensin, oestriol |
| | MR-55 | Lung biopsy | 5 | | | ACTH, bombesin, calcitonin |
| | MR-86 | Pleural fluid | 4 | 1–2 | 7 | — |
| | MR-103 | Pleural fluid | 3–5 | 3–5 | 14–28 | bombesin, calcitonin, estriol |
| Squamous carcinoma | MR-9 | Lung biopsy | 1,5 | | | ACTH, bombesin, neurotensin |
| | MR-25 | Lung biopsy | 1 | 20 | 80 | ACTH, bombesin, calcitonin, neurotensin, substance P |
| | MR-32 | Lung biopsy | 1,5 | 6–12 | 70 | bombesin, calcitonin, substance P |
| | MR-65 | Lung biopsy | 1 | 4–7 | 56 | bombesin, calcitonin, neurotensin |
| | MR-90 | Lung biopsy | 1 | 7–10 | 100 | — |
| Adeno-carcinoma | MR-5 | Lung biopsy | 1–3 | 20 | 140 | ACTH, bombesin, β-lipotropin |
| | MR-13 | Lung biopsy | 6 | 24 | 120 | ACTH, bombesin, calcitonin neurotensin |
| Large cell carcinoma | MR-8 | Lung biopsy | 3 | 6–10 | 35–42 | ACTH, bombesin, calcitonin, β-lipotropin, neurotensin, oestriol |
| | MR-97 | Lung biopsy | 1 | 7–18 | 56–70 | ACTH, bombesin, neurotensin |

[a]All hormones described in the text were assayed in the culture medium of each cell line. Hormones which are not described were not detectable by the applied methods.

Adherent growing small cell lung carcinoma cell lines exhibited growth characteristics of the suspension culture of cell aggregates after transplantation into the nude mouse. Between the second and the fifth passage of the reestablished cell culture the aggregates attached to the surface of the culture flask and changed to an adherent growth behaviour.

### *Morphological examinations of the cell lines*

The histological identity of the cells growing in permanent cell culture and their heterotransplants with the original human tumour was assisted by light and electron microscopic examination.

In the following section representative fine structural details of each type of tumour as established in xenografts and tissue culture will be summarized. Adenocarcinomas were mainly of the acinar type and preserved all fine structural characteristics of the primary tumour in all passages of xenotransplantation. The tumour cells contained abundant rough endoplasmic reticulum (RER), elaborate Golgi complexes and numerous electron translucant secretory granules (Figure 1a), which at the light microscopical level were identified as mucin granules. On their luminal surface the tumour cells projected numerous microvilli into the tubular lumen and revealed signs of exocytotic discharge of mucin granules (Figure 1a). Most of these characteristics, such as surface microvilli, RER, mucin granules were also preserved in cell culture (Figure 1b, c). In addition the tumour cells contained small dense core granules, resembling hormone storage granules in other cell types (Figure 1d). Xenografts of squamous cell carcinoma grew in a stratified pattern (Figure 2a), with intercellular bridges and occasional signs of tonofilament bundles (Figure 2b). In tissue culture the tumour cells grew partly in epithelial contact but formation desmosomes and tonofilaments was minimal or lacking (Figure 2c). These tumour cells contained predominantly free ribosomes, occasional profiles of RER and a few dense core vesicles, mainly in the peripheral part of the cytoplasm (Figure 2d). Tumour cells from small cell carcinoma were mainly obtained from pleural exudates and the structural identity between primary tumour, xenograft and tissue culture was greatest among the four types of tumour studied (Figure 3a). While in primary tumour cells dense core granules were mainly concentrated in pseudopodlike processes of the cells (Figure 3b) xenografted tumour cells contained similar granules both in the cytoplasm and in cytoplasmic processes (Figure 3c). In tissue culture the storage of such

**Figure 1** Fine structural characteristics of lung adenocarcinoma after xenotransplantation into nude mice (a) and growth in cell culture (b). Xenotransplanted tumour cells grow as tubular structures and contain elaborate RER and secretion granules which are discharged at the luminal surface (a, inset). In cell culture tumour cells reveal granules of two sizes: one measuring 0.8 –1.0 μm in diameter containing flocculent material (c) and dense core granules measuring 0.2 μm in diameter (d). Magnification: (a) ×2850, inset ×30000; (b) ×60000; (c)+(d) ×12000.

**Figure 2** Squamous cell carcinoma of the lung grown in nude mice (a, b) and in cell culture (c, d). Xenografted tumours preserve the structural characteristics of the original tumour and show bundles of tonofilament in association with desmosomes (arrows in b). In culture the cells grow as large undifferentiated epithelial cells with occasional dense core vesicles (arrow in d). Magnification: (a)+(c) ×2850; (b)+(d) ×12000.

**Figure 3** Fine structure of small cell carcinoma (a) tumour cells collected from pleural exudate (b) primary tumour cells at higher magnification. Note the occasional dense core vesicles in cytoplasm (arrow heads) and their concentration in pseudopodlike processes (arrows).

The structural characteristics are preserved when tumour cells are xenografted (c) or grown in cell culture (d). In xenografts (c) dense core vesicles are observed both in the cycloplasm and in pseudopod-like processes (arrows). Magnification: (a) ×2850; (b) ×12000; (c) ×12000; (d) ×12000.

granules was less pronounced, well developed profiles of RER and elaborate Golgi complexes indicated biosynthesis of exportable proteins (Figure 3d). Large cell carcinomas were characterised by tumour cells with large nuclei, prominent nucleoli and a dense network of intermediate filaments in the cytoplasm (Figure 4a, b). These features and the compact growth pattern of the tumour cells were well preserved after xenotransplantation.

### *Secretion of peptide hormones by lung tumour cells in culture*

ACTH, bombesin, calcitonin, and neurotensin immunoreactive proteins were demonstrated in the culture medium of lung tumour cell lines deriving from all four histological types. In additional to these peptides the cell culture media were analyzed for CEA, oestriol, β-HCG, β-lipotropin, substance P, ferritin, CA 19-9™, cortisol, progesterone, testosterone and aldosterone. For evaluation of each assay base line the hormones were determined diluted in fresh, 3, 5, 10, and 20 day old (incubation at 37°C) cell culture medium. Moreover, all hormones were assayed in the presence of culture medium from human fibroblasts, macrophages and lymphocytes. Determinations with inter-assay variations >15% were discarded. Observations on both primary and permanent cell cultures yielded the following data: positive ACTH levels were found in 31% ($n=16$) of the cell cultures deriving from small cell, 30% ($n=10$) from large cell, 24% ($n=29$) from squamous, and 20% ($n=13$) from adeno carcinoma of the lung. Bombesin production was observed in 50% ($n=16$) of small cell, 60% ($n=10$) of large cell, 63% ($n=29$) of squamous, and 46% ($n=13$) of the adeno lung tumour cell cultures. Elevated calcitonin levels were demonstrated in culture media of 43% ($n=16$) of small cell, 50% ($n=10$) of the large cell, 20% ($n=29$) of squamous, and 39% ($n=13$) of the adeno carcinoma cell cultures. Neurotensin positivity was determined in 25% ($n=12$) of the small cell, 40% ($n=10$) of the large cell, 20% ($n=25$) of the squamous, and 30% ($n=10$) of the adeno lung tumour cell cultures.

Immunological reactivity to some of the other proteins or steroids examined was observed only in some cell culture supernatants of small cell as well as non small cell lung tumour cell lines. Substance P was detectable in 20% ($n=31$), oestriol in 29% ($n=31$) and CA 19-9™ in 26% ($n=15$) of all culture media independent of the histological type of the original human tumour. In the case of oestriol it is also possible that there was no *de novo* steroid hormone biosynthesis but a conversion of a pecursor steroid present in the foetal calf serum. Table I summarizes the biosynthetic products of permanent lung tumour cell lines in relation to the

**Figure 4** Large cell carcinoma after xenotransplantation in nude mice. Note presence of dense core granules in the cytoplasm (a) and in pseudopod-like processes (b). Magnification: (a) ×2850; (b) ×6000.

**Figure 5** Peptide hormone levels in culture medium of permanent lung cancer cell lines. (△) ACTH; (□) bombesin; (●) calcitonin.

histological origin of the cell. The concentration of ACTH, calcitonin and bombesin immunoreactive proteins in the cell culture medium of permanent cell lines deriving from four lung tumour histologies were similar to those of primary C-cell carcinoma cell lines cultured under the same conditions (Figure 5).

### Discussion

The vast majority of bronchogenic carcinomas can be classified into 4 major histological types (Kreyberg *et al.*, 1967). On account of their histological characteristics these are classified into two groups: small cell lung tumours and non small cell lung tumours (Gazdar *et al.*, 1981). Small cell carcinoma of the lung is the most common non-endocrine tumour that is associated with the production of a variety of hormones (Group *et al.*, 1980). However, until now neither the incidence nor the spectrum of hormone production by small cell lung tumour cells have been clearly elucidated. Frequently multiple hormones have been demonstrated in the same tumour (Sorenson *et al.*, 1981). Nevertheless a number of examinations *in vivo* and *in vitro* have improved our knowledge of the biology of the small cell lung tumour type, while our information on non small cell lung carcinomas is limited.

ACTH immunoractivity has been identified in small cell non small cell lung tumours *in vivo* and *in vitro*. There is evidence that these peptides purified from small cell lung tumour cells are corticotropin-$\beta$-lipotropin precursor molecules (Luster *et al.*, 1983). Elevated calcitonin levels found in the sera especially of small cell lung tumour patients are derived not only from tumour cells but from other tissues as well (Roos *et al.*, 1980). Our results show (Luster *et al.*, 1982) that elevated calcitonin levels observed *in vivo* are caused by an ectopic hormone production by the tumour.

Moody *et al.* (1981) described biosynthesis of bombesin only by small cell lung tumour cells cultured *in vitro*. On the basis of these and some other biological criteria of small cell lung tumours it was suggested that these tumour cells might be derived from pulmonary endocrine cells, the Kultschitzky-like cells in the bronchial submucosa (Skrabanek & Powell, 1978). Immunohistological demonstration of calcitonin and other peptide hormones in non small cell as well as small cell lung tumour cells was in contradiction of this concept (Gropp *et al.*, 1981, Luster *et al.*, 1982). Permanent tumour cell cultures deriving from lung tumours of the 4 major histological types were established to ascertain whether all lung tumours produce a variety of peptide hormones. Based on a large number of non small cell lung tumour cell lines, the growth behaviour and peptide hormone biosynthesis of all 4 lung tumour types were examined. Bombesin, for instance, was demonstrated in cell culture media of >60% of the total number of 52 primary and long term tumour cell cultures of non small cell origin. Accordingly the biosynthesis of bombesin seems to be characteristic for all four major lung tumour types. From the wide spectrum of hormones, including even steroid hormones demonstrated in cell cultures from small cell as well as from non small cell lung tumours, it can be concluded that the biosynthesis of hormone immunoreactive proteins might be a common ability of all lung tumour types. In 5 to 35% of all bronchogenic carcinomas, the histology is mixed. It is not yet clear if these tumours are stages of conversion from one tumour type to the other or if they originate from two different tumour stem cells (Gazdar *et al.*, 1981; McDowell *et al.*, 1982).

Using light microscopical techniques the lack of specific markers makes it impossible to identify unequivocally the various types of cells growing in cell culture. It might be concluded that synthesis of peptide hormones is derived from some small cells which were already present in a mixed population within the primary tumour or that small cells differentiate to non small cells *in vitro*. To exclude these possibilities, xenografts have been established from permanent cell cultures to compare them with the original tumour specimen from which the cell culture was started (Shimosato *et al.*, 1976; Pettengill *et al.*, 1980). A close histological identity of the xenotransplants with the histology of the primary tumour was demonstrated. Accordingly, the cell lines which synthesize peptide hormones consist of cells belonging to the histological type of the original tumour. In agreement with Carney *et al.* (1983) we found that tumour cell cultures re-established from xenotransplants did not differ from cell cultures directly grown from patient tumour specimens.

The great differences in hormone production between small cell and non small cell bronchial carcinomas observed *in vivo,* which are in contrast to our *in vitro* results, may be partially explained by the different proliferation behaviour of small cell and non small cell lung tumours *in vivo* and *in vitro*. The non small cell lung tumour cells in culture in most cases exhibited a higher proliferation rate than cells originating from small cell lung carcinomas.

This is possibly due to non optimal conditions for small cell lung tumour cells in fluid cultures. In contrast, the clinical course of the small cell lung tumour is characterized by the highest growth rate of all lung tumour types. Xenotransplants established from slowly proliferating small cell lung tumour cell lines show rapid development similar to that of the solid tumours. Another reason for the observed *in vivo* and *in vitro* differences in the hormone concentrations in the periphery of the tumours may be the variable degradation of the hormones. Bombesin, for instance, and some other small peptides are characterized by a very short half-life *in vivo*. Nevertheless, such unstable hormones may play an important role in tumour regulation (Roth *et al.,* 1982). Gazdar *et al.* (1980) and Sherwin *et al.* (1981) recently reported studies on lung tumour cell lines which produced diffusable "growth factors". Pseudopod-like contacts between tumour cells observed ultrastructurally may be interpreted as a morphological hint for paracrine regulation in the tumour tissue.

It can be concluded that *in vivo* differences in hormone biosynthesis between small cell and non small cell lung tumours seem to be a quantitative not a qualitative phenomenon. It is apparent that lung cancers present a continuous spectrum of tumour types which may have a common cellular origin. The importance of the universal characteristics of hormone production by cells of all 4 lung tumour histologies may be understood in the autocrine or paracrine regulation of growth or differentiation of these tumours. Recent findings indicate that hormonal polypeptides involved in intercellular communication arose very early in evolution, even in prokaryotes, and have been highly conserved up to man (Roth *et al.,* 1982).

### References

BARCK, H.C. (1982). *Histologische Technik*. Thieme New York, p. 148.

BERSON, S.A. & YALOW, R.S. (1966). Parathyroid hormone in plasma in adenomatous hyperparathyroidism, uremia and bronchogenic carcinoma. *Science,* **153,** 907.

CARNEY, D.N., BRODER, L., EDELSTEIN, M. & 6 others. (1983). Experimental studies in the biology of human small cell lung cancer. *Cancer Treat. Rep.,* **67,** 27.

GAZDAR, A.F., CARNEY, D.N., RUSSELL, E.K. & 5 others. (1980). Establishment of continuous clonable cultures of small cell carcinoma of the lung which have amine precursor uptake and decarboxylation cell properties. *Cancer Res.,* **50,** 3502.

GAZDAR, A.F., CARNEY, D.N., GUCCION, J.G. & 4 others. (1981). Small cell carcinoma of the lung: Cellular origin and relationship to other pulmonary tumours. In: *Small Cell Lung Cancer*. (Eds. Greco *et al.*), New York: Grune & Stratton, p. 145.

GROPP, C., HAVEMANN, K. & SCHEUER, A. (1980). Ectopic hormones in lung cancer patients at diagnosis and during therapy. *Cancer,* **46,** 347.

GROPP, C., SOSTMANN, H., LUSTER, W., KALBFLEISCH, H., LEHMANN, F.-G. & HAVEMANN, K. (1981). ACTH, β-Lipotropin, β-Endorphin, β-HCG, Calcitonin and CEA in Lung Tumor Tissues. In: *CEA und andere Tumormarker*. (Eds. Uhlenbruck & Wintzer), Tumor-Diagnostik-Verlag Leonberg, p. 217.

HENNING, N. (1966). *Klinische Laboratoriumsdiagnstik*. Urban und Schwarzenberg, München, Wien p. 149.

HORAI, T., NISHIHARA, H. TATEISHI, R., MATSUDA, M. & HATTORI, S. (1973). Oat-cell carcinoma of the lung simultaneously producing ACTH and serotonin. *J. Clin. Endocrinol. Metab.,* **27,** 212.

KREYBERG, L., LIEBOW, A.A. & UEHLINGER, E.A. (1967). *Histological Typing of Lung Tumours*. World Health Organisation, Geneva.

LUSTER, W., GROPP, C. & HAVEMANN, K. (1983). Peptide hormone synthesizing lung tumour cell lines: Establishment and first characterization of biosynthetic products. *Acta Endocrinol. (Supp),* **253,** 24.

LUSTER, W., GROPP, C., SOSTMANN, H., KALBFLEISCH, H. & HAVEMANN, K. (1982). Demonstration of immunoreactive calcitonin in sera and tissues of lung cancer patients. *Eur. J. Cancer Clin. Oncol.,* **18,** 1275.

McINTOSH, C., ARNOLD, R., BOTHE, E., BECKER, H., KÖBBERLING, J. & CREUTZFELD, W. (1978). Gastrointestinal somatostatin: extraction and radioimmunoassay in different species. *GUT,* **19,** 655.

McDOWELL, E.M., HARRIS, C.C. & TRUMP, B.F. (1982). Histogenesis and morphogenesis of bronchogenc neoplasms. In: *Morphogenesis of Lung Cancer*. (Eds. Shimato *et al.*) Boca Raton: Vol. 1 CRC Press, 1.

MOODY, T.W., PERT, C.B., GAZDAR, A.F. & MINNA, J.D. (1981). High levels of intracellular bombesin characterize human small-cell lung carcinoma. *Science,* **214,** 1246.

PETTENGILL, O.S., CURPHEY, T.J., CATE, C.C., FLINT, C.F., MAURER, L.H. & SORENSON, G.D. (1980). Animal model for small cell carcinoma of the lung; effect of immunosuppression and sex of mouse on tumour growth in athymic nude mice. *Exp. Cell. Biol.*, **48**, 279.

REYNOLDS, E.S. (1963). The use of lead citrate at high pH as an electron opaque stain in electron microscopy. *J. Cell. Biol.*, **17**, 208.

ROTH, J., LeROITH, D., SHILOACH, J., ROSENZWEIG, J.L., LESNIAK, M.A. & HAVRANKOVA, J. (1982). The evolutionary origins by hormones, neurotransmitters, and other extracellular chemical messengers. *N. Engl. J. Med.*, **306**, 523.

ROOS, B.A., LINDALL, A.W., BAYLIN, S.B. & 4 others. (1980). Plasma immunoreactive calcitonin in lung cancer. *J. Clin. Endocrinol. Metab.*, **50**, 659.

SHERWIN, S.A., MINNA, J.D., GAZDAR, A.F. & TODARO, G.J. (1981). Expression of epidermal and nerve growth factor receptors and soft agar growth factor production by human lung cancer cells. *Cancer Res.*, **41**, 3538.

SHIMOSATO, Y., KAYEMA, T., TVAGI, K. & 4 others. (1976). Transplantation of human tumours into nude mice. *J. Natl Cancer Inst.*, **56**, 1251.

SILVA, O.L., BECKER, K.L., PRIMACK, A., DOPPMAN, I. & SNIDER, R.H. (1974). Ectopic section of calcitonin by oat-cell carcinoma. *N. Engl. J. Med.*, **290**, 1122.

SILVA, O.L., BRODER, L.E., DOPPMAN, J.L. & 4 others. (1979). Calcitonin as a marker for brochogenic cancer. *Cancer*, **44**, 680.

SKRABANEK, P. & POWELL, D. (1978). Unifying concept of non pituitary ACTH secreting tumours. Evidence of common origin of neural crest tumours, carcinoids, and oat-cell carcinomas. *Cancer*, **42**, 1263.

SORENSON, G.D., PETTENGILL, O.S., BRINCK-JOHNSON, T., CATE, C.C. & MAURER, L.H. (1981). Hormone production by cultures of small cell carcinoma of the lung. *Cancer*, **47**, 1289.

TAKAHASI, M. (1981). *Color Atlas of Cancer Cytology*. Thieme Verlag, Stuttgart/New York.

WOLFSEN, A.R. & ODELL, W.D. (1979). ProACTH: use for early detection of lung cancer. *Am. J. Med.*, **66**, 765.


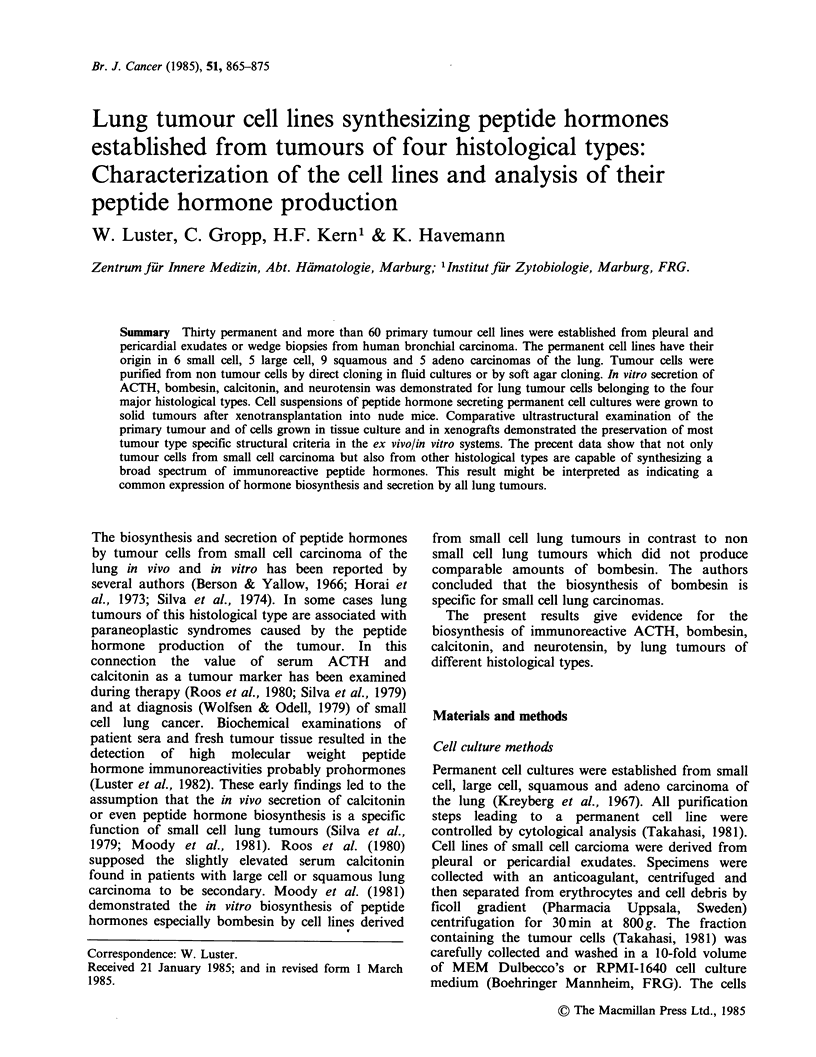

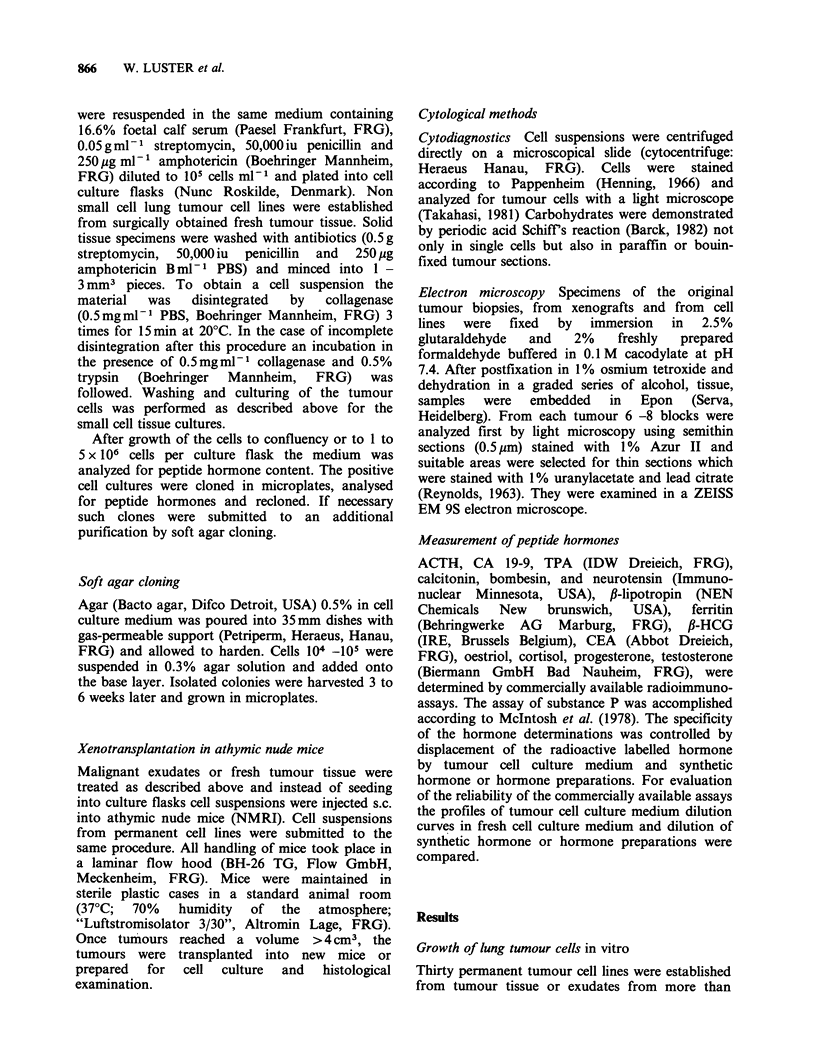

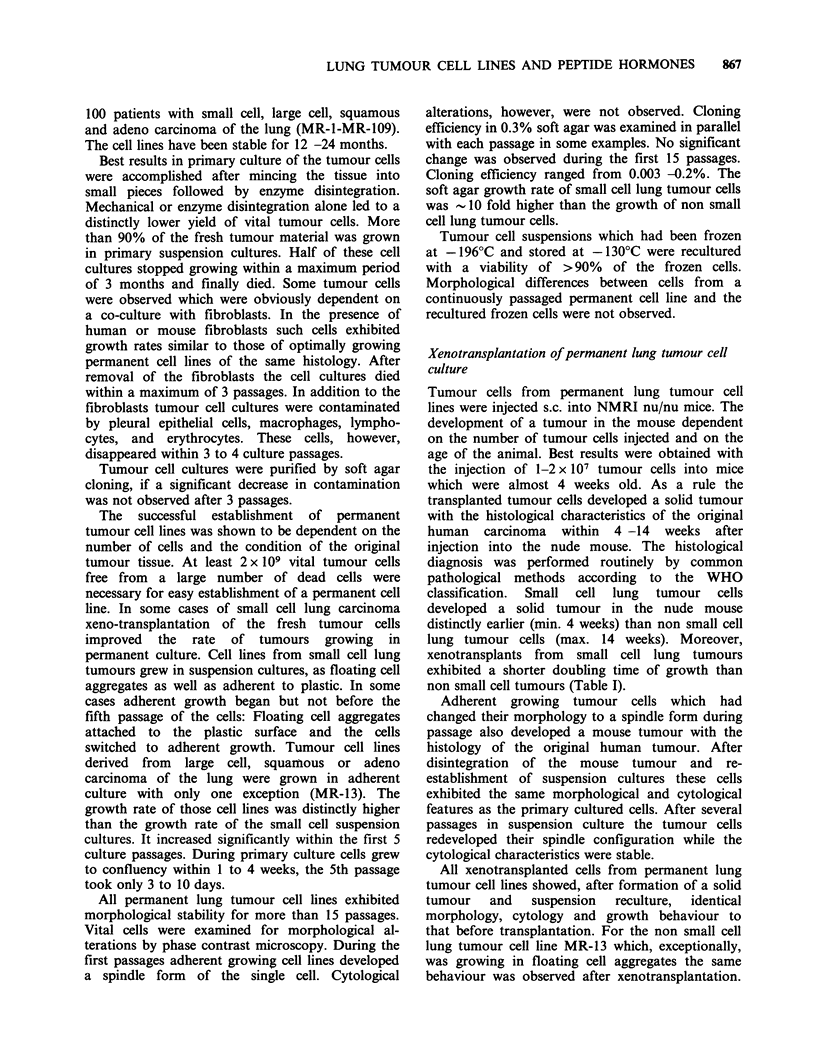

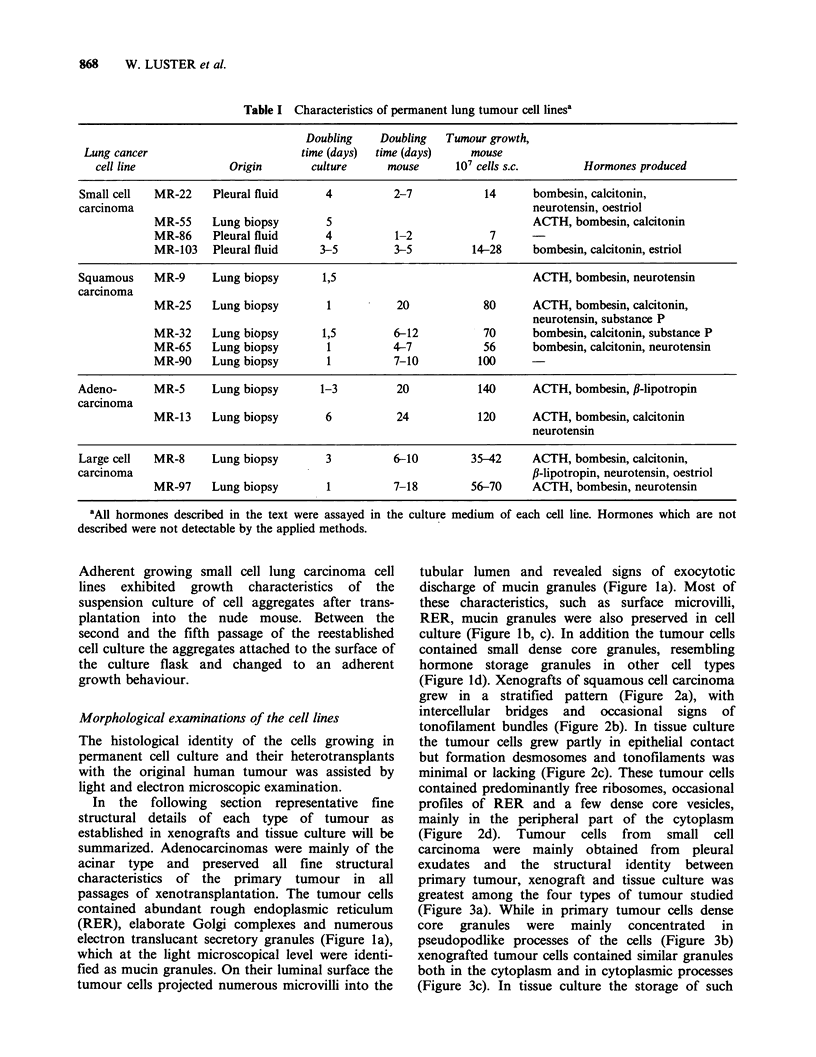

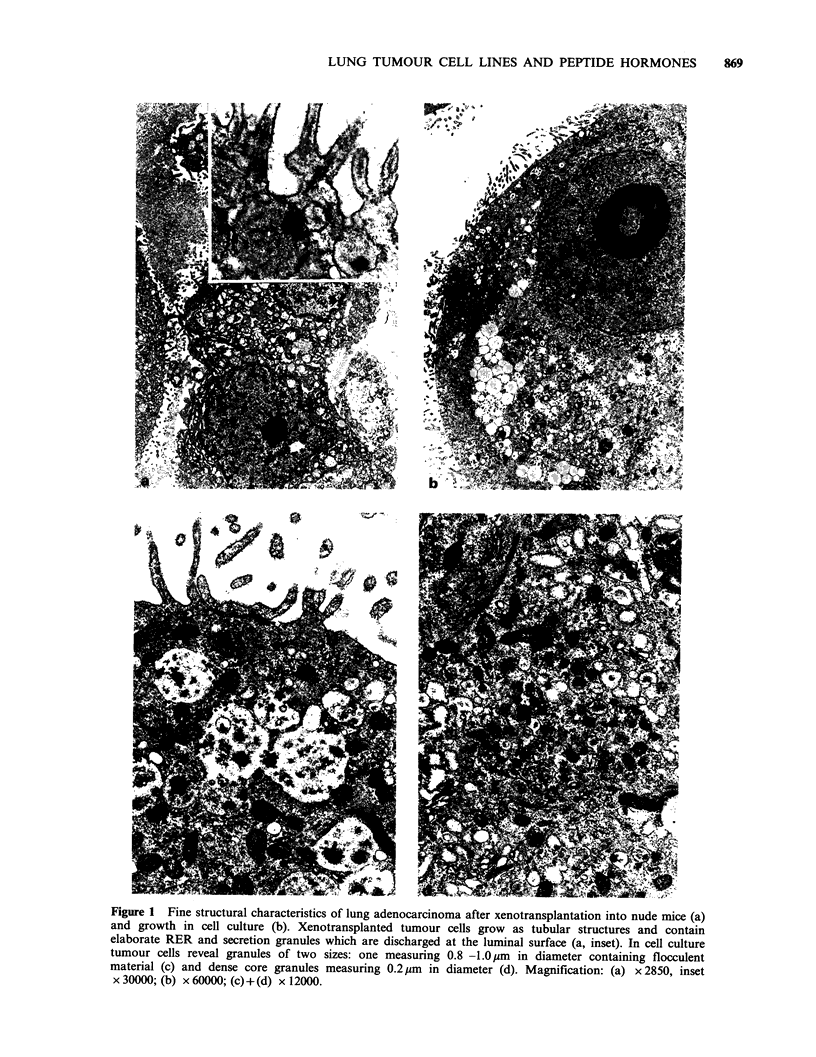

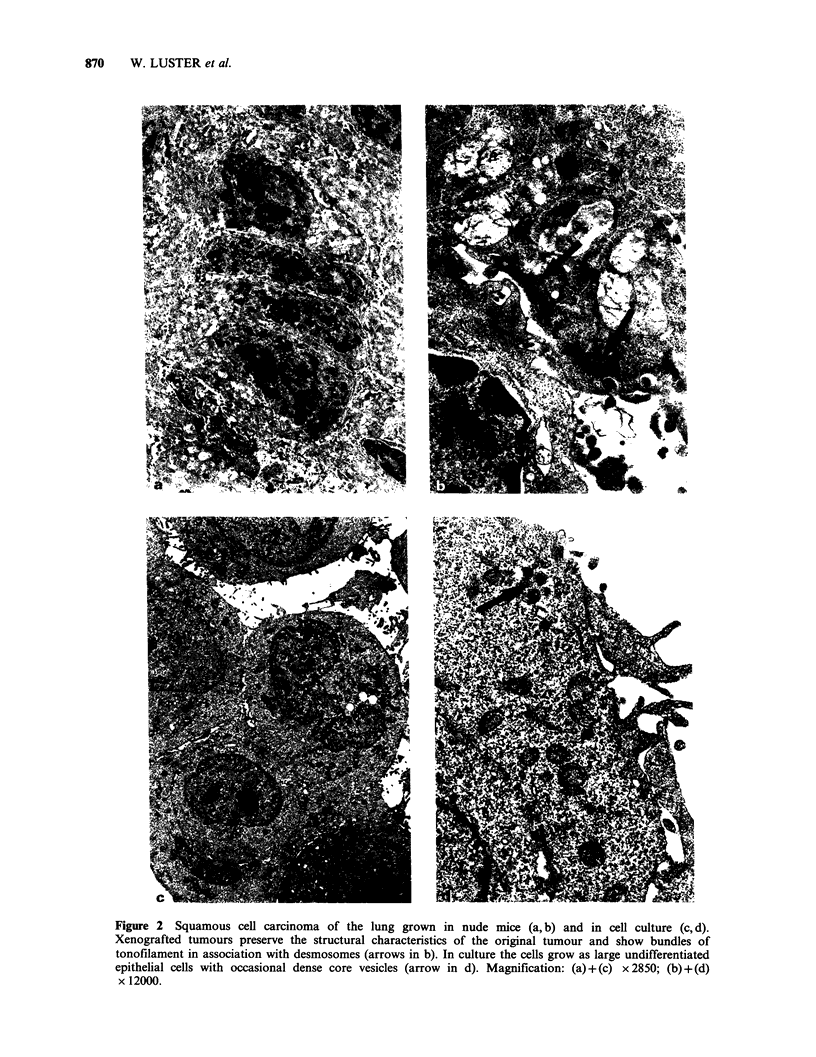

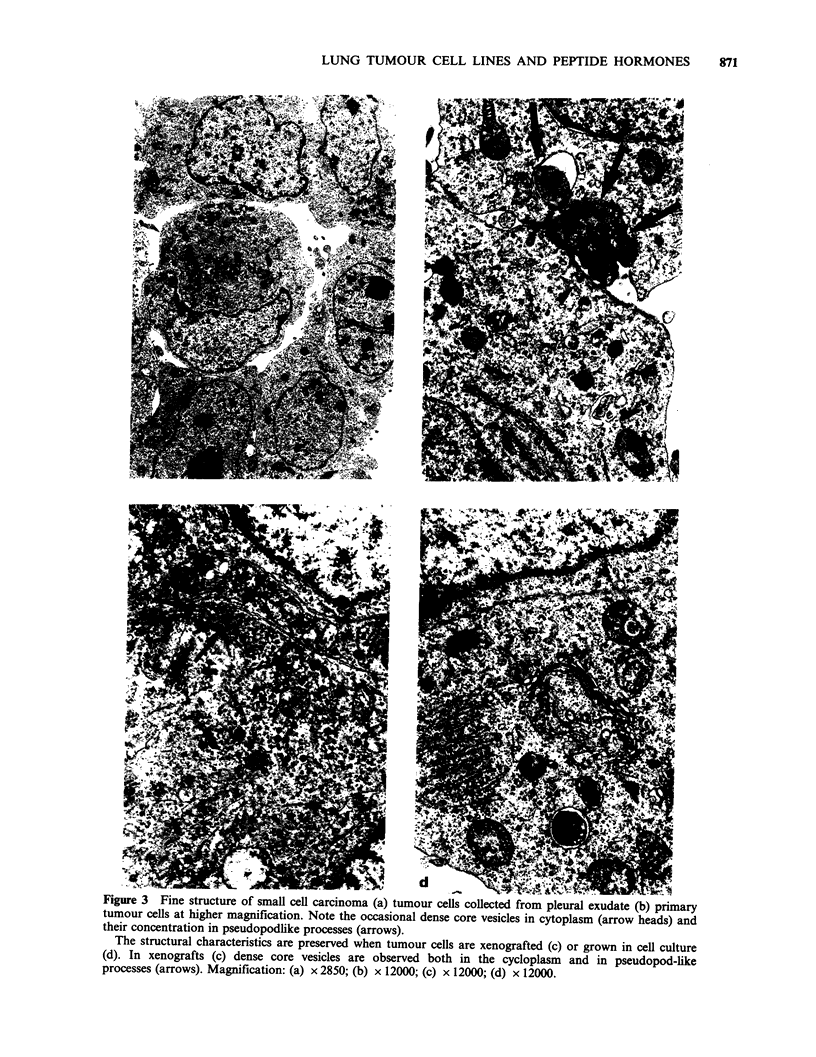

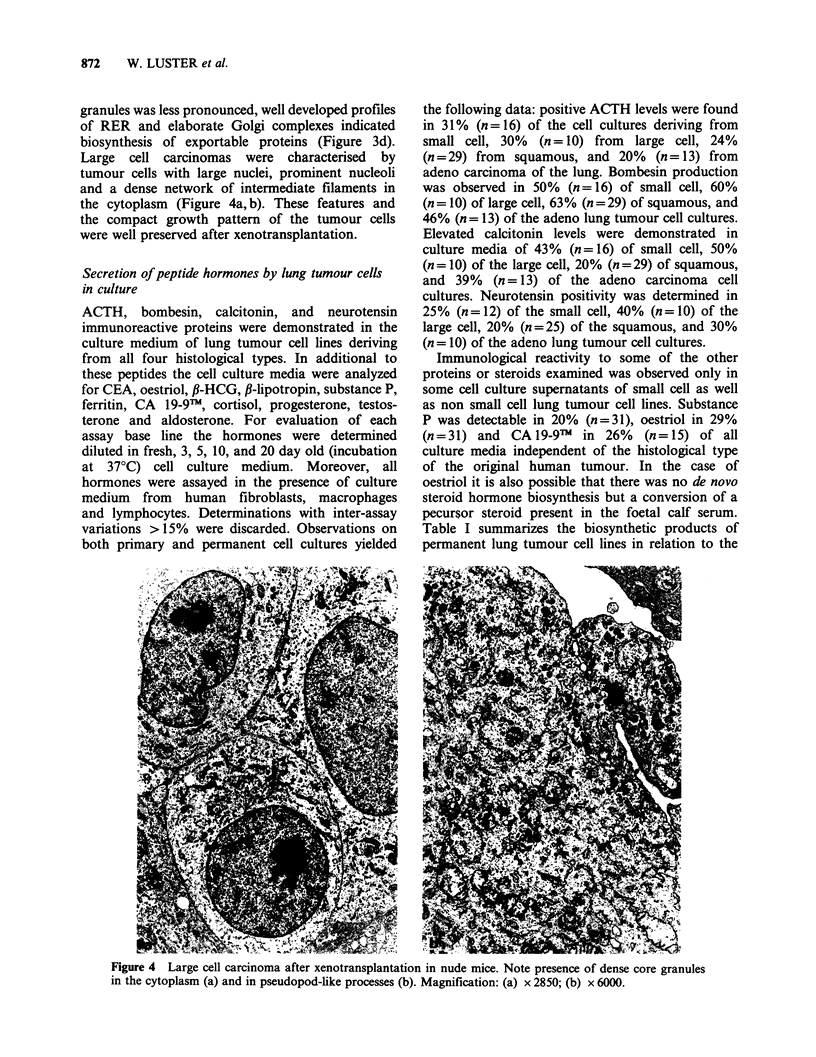

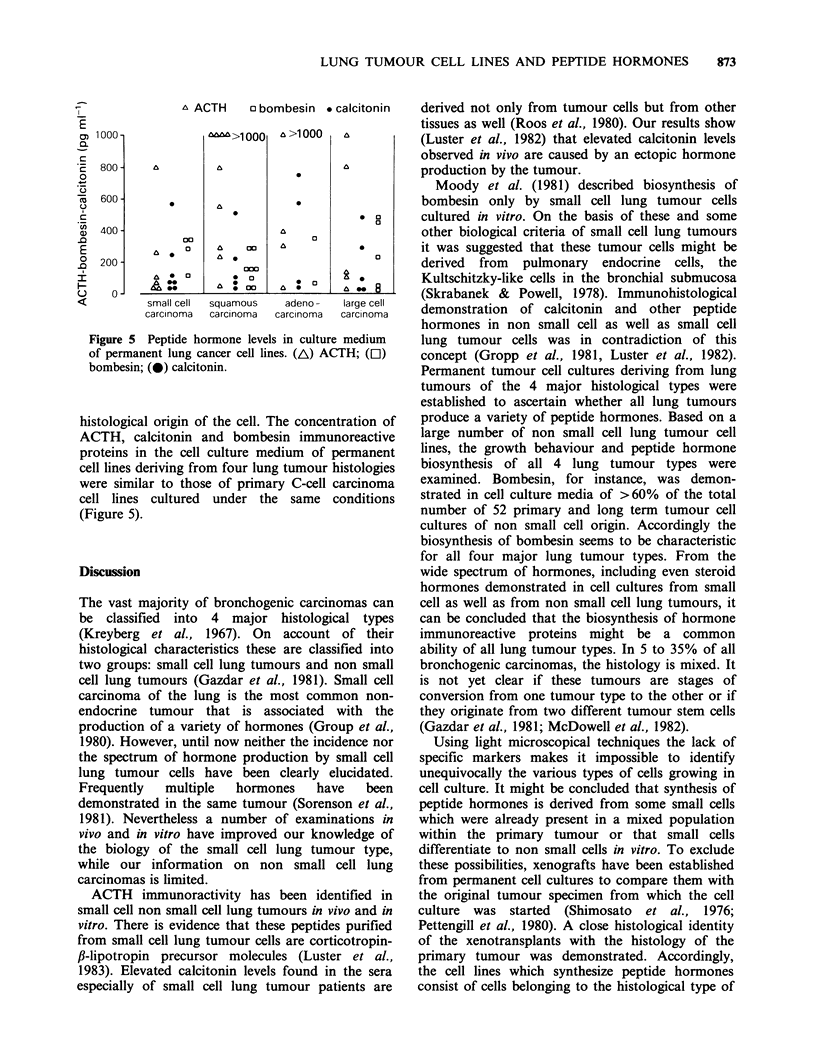

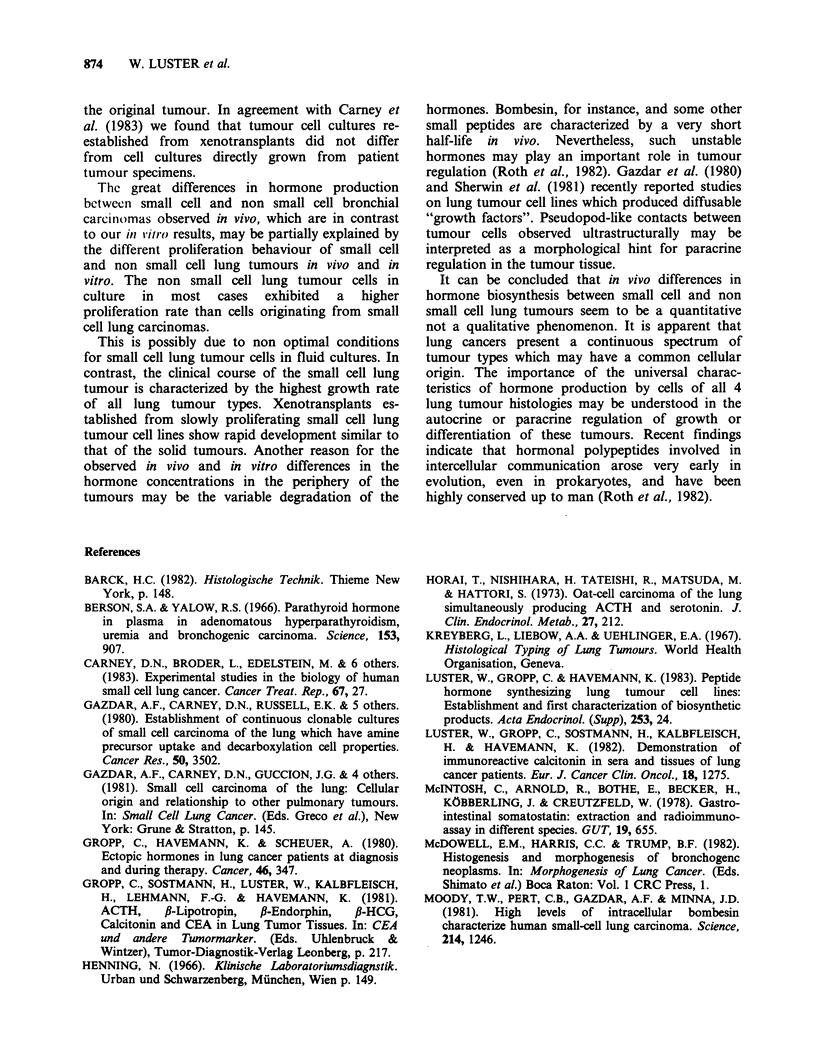

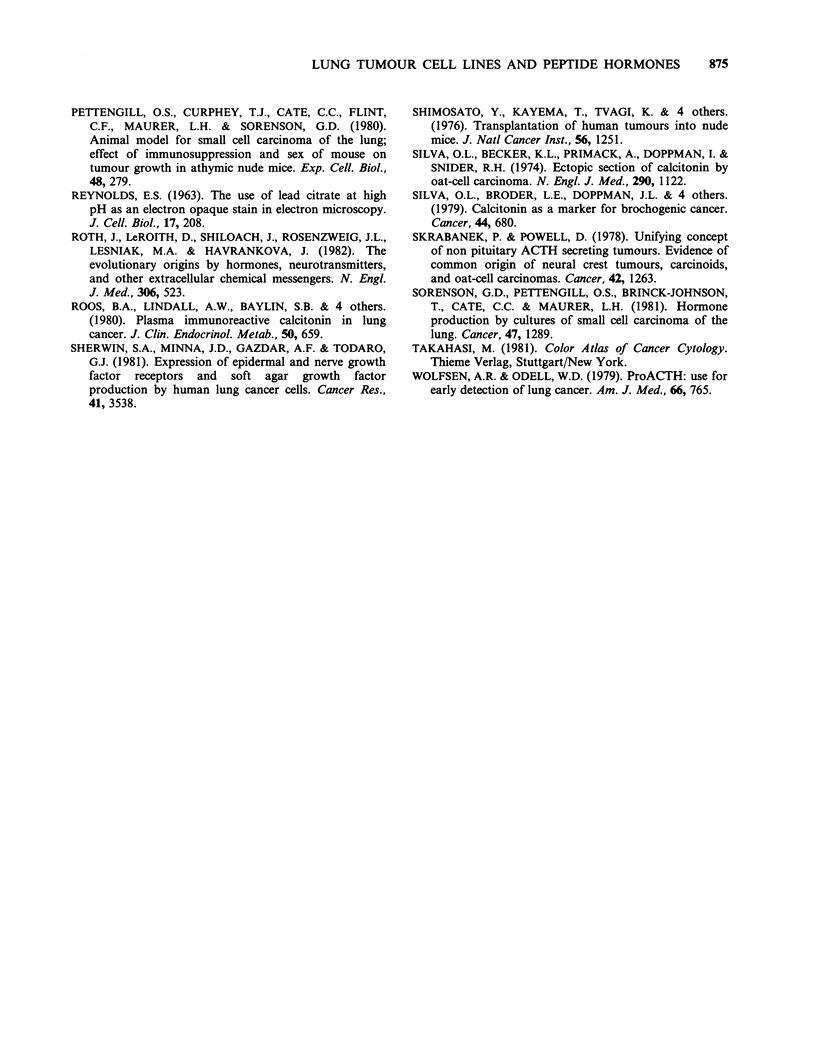


## References

[OCR_00775] Berson S. A., Yalow R. S. (1966). Parathyroid hormone in plasma in adenomatous hyperparathyroidism, uremia, and bronchogenic carcinoma.. Science.

[OCR_00786] Gazdar A. F., Carney D. N., Russell E. K., Sims H. L., Baylin S. B., Bunn P. A., Guccion J. G., Minna J. D. (1980). Establishment of continuous, clonable cultures of small-cell carcinoma of lung which have amine precursor uptake and decarboxylation cell properties.. Cancer Res.

[OCR_00800] Gropp C., Havemann K., Scheuer A. (1980). Ectopic hormones in lung cancer patients at diagnosis and during therapy.. Cancer.

[OCR_00817] Horai R., Nishihara H., Tateishi R., Matsuda M., Hattori S. (1973). Oat-cell carcinoma of the lung simultaneously producing ACTH and serotonin.. J Clin Endocrinol Metab.

[OCR_00834] Luster W., Gropp C., Sostmann H., Kalbfleisch H., Havemann K. (1982). Demonstration of immunoreactive calcitonin in sera and tissues of lung cancer patients.. Eur J Cancer Clin Oncol.

[OCR_00840] McIntosh C., Arnold R., Bothe E., Becker H., Köbberling J., Creutzfeldt W. (1978). Gastrointestinal somatostatin: extraction and radioimmunoassay in different species.. Gut.

[OCR_00852] Moody T. W., Pert C. B., Gazdar A. F., Carney D. N., Minna J. D. (1981). High levels of intracellular bombesin characterize human small-cell lung carcinoma.. Science.

[OCR_00860] Pettengill O. S., Curphey T. J., Cate C. C., Flint C. F., Maurer L. H., Sorenson G. D. (1980). Animal model for small cell carcinoma of the lung. Effect of immunosuppression and sex of mouse on tumor growth in nude athymic mice.. Exp Cell Biol.

[OCR_00868] REYNOLDS E. S. (1963). The use of lead citrate at high pH as an electron-opaque stain in electron microscopy.. J Cell Biol.

[OCR_00880] Roos B. A., Lindall A. W., Baylin S. B., O'Neil J. A., Frelinger A. L., Birnbaum R. S., Lambert P. W. (1980). Plasma immunoreactive calcitonin in lung cancer.. J Clin Endocrinol Metab.

[OCR_00873] Roth J., LeRoith D., Shiloach J., Rosenzweig J. L., Lesniak M. A., Havrankova J. (1982). The evolutionary origins of hormones, neurotransmitters, and other extracellular chemical messengers: implications for mammalian biology.. N Engl J Med.

[OCR_00885] Sherwin S. A., Minna J. D., Gazdar A. F., Todaro G. J. (1981). Expression of epidermal and nerve growth factor receptors and soft agar growth factor production by human lung cancer cells.. Cancer Res.

[OCR_00897] Silva O. L., Becker K. L., Primack A., Doppman J., Snider R. H. (1974). Ectopic secretion of calcitonin by oat-cell carcinoma.. N Engl J Med.

[OCR_00902] Silva O. L., Broder L. E., Doppman J. L., Snider R. H., Moore C. F., Cohen M. H., Becker K. L. (1979). Calcitonin as a marker for bronchogenic cancer: a prospective study.. Cancer.

[OCR_00907] Skrabanek P., Powell D. (1978). Unifying concept of non-pituitary ACTH-secreting tumors: evidence of common origin of neural-crest tumors, carcinoids, and oat-cell carcinomas.. Cancer.

[OCR_00913] Sorenson G. D., Pettengill O. S., Brinck-Johnsen T., Cate C. C., Maurer L. H. (1981). Hormone production by cultures of small-cell carcinoma of the lung.. Cancer.

[OCR_00923] Wolfsen A. R., Odell W. D. (1979). ProACTH: use for early detection of lung cancer.. Am J Med.

